# Activated PI3 Kinase Delta Syndrome: From Genetics to Therapy

**DOI:** 10.3389/fimmu.2018.00369

**Published:** 2018-02-27

**Authors:** David Michalovich, Sergey Nejentsev

**Affiliations:** ^1^Refractory Respiratory Inflammation Discovery Performance Unit, GlaxoSmithKline, Stevenage, United Kingdom; ^2^Department of Medicine, University of Cambridge, Cambridge, United Kingdom

**Keywords:** activated PI3 kinase delta syndrome, primary immunodeficiency, phosphoinositide-3-kinase δ, mutation, inhibitor

## Abstract

Activated PI3 kinase delta syndrome (APDS) is a primary immunodeficiency caused by dominant mutations that increase activity of phosphoinositide-3-kinase δ (PI3Kδ). APDS can be caused by mutations in the *PIK3CD* gene that encodes PI3Kδ catalytic subunit p110δ (APDS1) or mutations in the *PIK3R1* gene that encodes regulatory subunit p85α (APDS2). APDS research advanced rapidly after the initial discovery in 2013. More than 200 APDS patients have been identified around the world. Multiple novel APDS mutations were reported and molecular mechanisms leading to PI3Kδ activation have been elucidated. The finding of APDS significantly increased our understanding of the role of PI3Kδ in the human immune system. Perhaps most importantly, discovery of the molecular basis of this primary immunodeficiency suggested that APDS patients, who previously received only non-specific therapy, could be treated by a novel class of drugs that inhibits PI3Kδ activity. This led to the ongoing clinical trials of selective PI3Kδ inhibitors in APDS patients. Overall, the APDS story provides an excellent example of translational research, beginning with patients who had an unknown disease cause and leading to a novel specific knowledge-based treatment.

## Introduction

Primary immunodeficiencies (PIDs) are a group of disorders that cause immune dysfunction and manifest with increased susceptibility to infections. Many PIDs are monogenic diseases. To date, mutations in more than 300 genes have been shown to cause various PIDs (1). Activated PI3 kinase delta syndrome (APDS) is a PID that results from gain-of-function mutations in genes encoding the phosphoinositide-3-kinase δ (PI3Kδ). This review will focus on the APDS mutations, phenotypes of the disease, and current therapeutic approaches.

Phosphoinositide-3-kinase δ is a class IA lipid kinase that phosphorylates phosphatidylinositol-4,5-bisphosphate [PtdIns(4,5)P_2_ or PIP_2_] to produce phosphatidylinositol-3,4,5-trisphosphate [PtdIns(3,4,5)P_3_ or PIP_3_]. There are three class IA PI3Ks in mammalian cells: α, β, and δ. Each class IA PI3K is composed of a catalytic subunit: p110α, p110β, or p110δ (encoded by genes *PIK3CA, PIK3CB*, and *PIK3CD*, respectively), and one of the five regulatory subunits: p85α, p55α, p50α (all encoded by different transcripts of the *PIK3R1* gene), p85β (encoded by the *PIK3R2* gene), or p55γ (encoded by the *PIK3R3* gene). The regulatory subunit stabilizes the catalytic subunit to prevent its proteasomal degradation, inhibits activity of the catalytic subunit, and recruits it to the plasma membrane (2). Catalytic subunits p110α and p110β are broadly expressed, while p110δ is mainly expressed in cells of the hematopoietic system, primarily lymphocytes and myeloid cells (3). In immune cells, PI3Kδ is activated downstream of cytokine receptors, toll-like receptors, B-cell and T-cell receptors, and Ras superfamily of small GTPases (4). PIP_3_ produced by PI3Ks activates kinases PDK1 and AKT, leading to the activation of mTOR complex 1 and inhibition of FOXO family of transcription factors. In lymphocytes, PIP_3_ activates kinases BTK and ITK that mediate activation of phospholipase Cγ and other proteins (3). PIP_3_ is dephosphorylated to PIP_2_ by a phosphatase PTEN.

## APDS Mutations

In 2013, two groups, one in Cambridge (UK) and the other in Bethesda (USA), used whole-exome-sequencing analysis of PID patients with unknown etiology and reported a novel PID caused by rare heterozygous germline gain-of-function mutations in the *PIK3CD* gene (5, 6). The mutations led to the increased PI3Kδ activity and the disease was called APDS (5) or p110δ-activating mutation causing senescent T cell, lymphadenopathy, and immunodeficiency (PASLI) (6) (OMIM #615513). Subsequently, rare heterozygous germline mutations in the *PIK3R1* gene were described that also resulted in an increased PI3Kδ activity and immune deficiency, phenocopying patients with the *PIK3CD* mutations. This disorder has been termed APDS2 or PASLI-R1 (7, 8) (OMIM #616005). Now, a PID caused by activating mutations in the *PIK3CD* gene is referred to as APDS1 and both diseases together are known as APDS.

**Figure 1:**
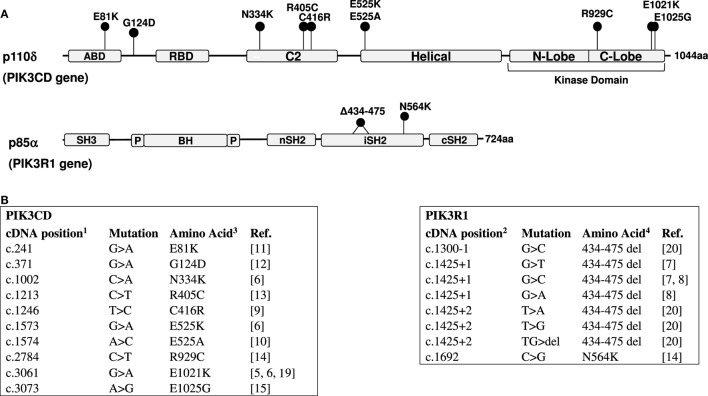
**(A)** Domain structure of the p110δ and p85α proteins and positions of mutations. ABD, adaptor-binding domain; RBD, Ras-binding domain; BH, breakpoint cluster region homology domain; P, proline-rich regions. **(B)** Activated PI3 kinase delta syndrome mutations in the *PIK3CD* (5, 6, 9–15, 19) and *PIK3R1* (7, 8, 14, 20) genes. 1—NM_005026; 2—NM_181523 (RefSeq); 3—O00329; 4—P27986 (UniProt).

Since the initial publications, 10 activating missense mutations have been reported in the *PIK3CD* gene resulting in APDS1 (5, 6, 9–15) (Figure 1). The E1021K variant in the C-lobe of the p110δ kinase domain is by far the most frequently reported APDS mutation. In the p110δ protein, E1021K is positioned similar to the somatic mutation H1047R of another PI3K isoform, p110α. Both E1021K and H1047R increase PI3K activity by enhancing association of the catalytic subunits with membranes and facilitating more effective phosphorylation of PIP_2_ (5, 16–18). The R929C mutation in the C-lobe of the p110δ kinase domain may also act in a similar manner (14). Other p110δ mutations located in the C2 domain (N334K, C416R) and the helical domain (E525K) likely interfere with inhibitory contacts between p110δ and p85α (18). Interestingly, activating somatic mutations of the homologous amino-acid residues of p110α (N345, C420, and E545) have been also found in tumors. The recently identified E81K and G124D mutations in the adapter-binding domain and the linker between the adapter-binding and the Ras-binding domains may affect the orientation of the adapter-binding domain and hence interaction between p110δ and p85α (11).

Several mutations causing APDS2 were identified in the *PIK3R1* gene (Figure 1). These include one missense mutation and seven mutations affecting the splice sites of exon 11 (coding exon 10), one affecting the splice acceptor site, and six affecting the splice donor site. All splice site mutations lead to the skipping of exon 11 and an in-frame deletion of 42 amino-acid residues in positions 434–475 within the inter-SH2 coiled-coil domain of p85α. The additional N564K variant in p85α is also found in the inter-SH2 domain (14). The inter-SH2 domain of p85α is known to inhibit the catalytic p110 subunit by interacting with its C2 domain (2). Interestingly, the APDS2 mutations in p85α lead to the disease that phenocopy APDS1, despite that p85α is ubiquitously expressed and interacts not only with p110δ but also with p110α and p110β. However, it has been demonstrated that the 42 amino-acid deletion in p85α effectively disrupts inhibitory interactions between p85α and p110δ, leading to a strong basal activation of PI3Kδ, while it only weakly increases PI3Kα activity (18). This differential effect explains why the impact of this mutation is largely restricted to the immune system.

Mutations that cause APDS have been found in patients from different countries around the world. So far, more than 200 APDS patients carrying activating mutations in the *PIK3CD* and *PIK3R1* genes have been identified. None of these variants were found in large cohorts of healthy subjects, e.g., they are absent from the largest human exome and whole-genome database gnomAD that includes more than 138,000 subjects (21). In several families, APDS mutations were shown to appear *de novo* among children, while being absent in their parents (5, 6, 10, 20), and long-range haplotype analysis in families with the E1021K mutation showed no founder effect (5). These findings indicate that APDS mutations appear recurrently in human populations. It is possible that activation of PI3Kδ provides selective advantages to cells during gametogenesis. Current data show that APDS mutations have high penetrance, e.g., out of the 53 subjects from 30 APDS1 families only one adult carrier of the E1021K mutation had no reported health issues (22). The true prevalence of APDS is not known. In the original study, heterogeneous cohorts comprising 184 PID patients were screened for the E1021K mutation and 17 APDS patients from seven unrelated families were identified (5). However, these cohorts included multiple patients with hyper-IgM syndrome and, therefore, were enriched for APDS mutations. Another study screened 669 patients with undefined PIDs for the N334K, C416R, E525K, and E1021K mutations in *PIK3CD* and the *PIK3R1* splice site mutations and found only *PIK3CD* mutations in three siblings diagnosed with common variable immune deficiency (CVID) and two sporadic cases with combined immunodeficiency (23). Thus, prevalence of APDS may vary considerably between different PID cohorts.

## APDS Phenotypes

Two comprehensive studies of APDS cohorts have been carried out recently and characterized its clinical and immunological manifestations (Table 1). One study examined the phenotypes of 53 patients with APDS1 (50 subjects with E1021K and 3 subjects E525K mutations) (22). The other studied 36 patients with APDS2 (20). Almost all APDS1 and APDS2 patients suffered from recurrent respiratory infections caused by bacterial pathogens, mainly *Streptococcus pneumoniae* and *Haemophilus influenzae*. Bronchiectasis was a common complication of lung infections affecting up to 60% of APDS1 patients. Interestingly, the majority of bronchiectasis patients had normal IgG levels and diagnosing PID in such subjects may not have been straightforward. Therefore, screening bronchiectasis patients without a clear PID for APDS mutations can reveal unrecognized APDS cases. Severe, persistent, or recurrent herpes virus infections, including EBV, CMV, HSV, and VZV infections, were found in 49% APDS1 and 31% APDS2 patients and were associated with lymphadenopathy. Immunologically, increased frequency of transitional B cells was often observed in APDS patients (Table 1). Many patients also had increased serum IgM levels and, therefore, some of the patients previous were diagnosed with hyper-IgM syndrome (5). Approximately one third of APDS1 patients and 17% of APDS2 patients had autoimmune or autoinflammatory manifestations. High incidence of lymphomas was also recorded in APDS patients (20, 22, 24). Unexpectedly, neurodevelopmental delay was found to be a relatively frequent manifestation in both APDS cohorts (Table 1), which may suggest an important role of PI3Kδ in the development of central nervous system that was not recognized previously.

**Table 1:** Characteristic clinical and immunological features of activated PI3 kinase delta syndrome (APDS).

Manifestations	APDS1 (22)	APDS2 (20)
Recurrent respiratory tract infections	96%	100%
Pneumonia	85%	71%
Bronchiectasis	60%	18%
Herpesvirus infections	49%	31%
Lymphadenopathy	64%	75%
Splenomegaly	58%	43%
Autoimmune or autoinflammatory disease	34%	17%
Neurodevelopmental delay	19%	31%
Lymphoma	13%	25%

Increased IgM	76%	58%
Increased transitional B cells^a^	75%	93%

*^a^If data were available and B cells were sufficient for analysis*.

Thus, APDS manifests as a PID with a high rate of recurrent respiratory tract infections, often leading to bronchiectasis, herpes virus infections, lymphadenopathy, splenomegaly, increased risk of lymphomas, frequent autoimmune manifestations, and, occasionally, developmental delay. In addition, APDS2 patients had a high frequency of growth retardation (45%), a feature that was not found in APDS1 patients. This difference may reflect impaired interactions of p85α with p110α and p110β catalytic subunits. Of note, a number of other dominant germline mutations, which reside within the nSH2 and iSH2 domains of p85α and reduce PI3K signaling, are known to cause the SHORT syndrome that includes short stature, hyperextensibility of joints, hernia, ocular depression, Rieger anomaly, and teething delay (25–28) (OMIM #269880). A single patient with a homozygous loss-of-function mutation in the *PIK3R1* gene was described that resulted in the absent p85α and reduced expression of p110δ. The patient had B lymphopenia and hypogammaglobulinemia and suffered from recurrent Campylobacter bacteremia and inflammatory bowel disease (29) (OMIM #615214). Also, a patient with biallelic loss-of-function mutations in the *PIK3CD* gene and reduced p110δ expression was reported to have B lymphopenia and hypogammaglobulinemia, sinopulmonary infections, septic arthritis, inflammatory bowel disease, and autoimmune hepatitis (30). Therefore, although p110δ deficiency also leads to a PID, its phenotype is different from APDS.

## Therapies for APDS Patients—Precision Medicine for a Rare Disease

Treatment regimes for APDS patients include antibiotic prophylaxis and immunoglobulin replacement therapy. Hematopoietic stem cell transplantation (HSCT) has been successful in several APDS patients and can be a treatment option, especially in young patients (20, 22). Immunosuppressive therapies aimed at reducing lymphoproliferation have included treatment with rituximab (anti-CD20 monoclonal antibody) and rapamycin to target the activation of the mTOR pathway. Treatment with rapamycin led to the improvement of immunological markers and a reduction in splenomegaly and lymphadenopathy (6). Nevertheless, the discovery of the APDS etiology and the causative role of mutations that activate PI3Kδ opened an opportunity for a novel specific treatment using selective PI3Kδ inhibitors. This class of drugs has been developed for cancer treatment (31), as well as inflammatory disorders, such as rheumatoid arthritis, asthma, and chronic obstructive pulmonary disease (COPD) (32–34). One of the PI3Kδ inhibitors, idelalisib, has been approved for treatment of chronic lymphocytic leukemia and non-Hodgkin lymphoma (35, 36). Idelalisib (previously known as GS-1101) reduced the catalytic activity of mutant PI3Kδ as efficiently as the activity of the wild type PI3Kδ (5, 18). PI3Kδ inhibitors also normalized PI3Kδ hyperactivation in cells of APDS patients *in vitro* (5–8, 37). These results opened way for clinical trials of PI3Kδ inhibitors in APDS patients.

Two phase-II clinical trials are currently ongoing to study the safety, pharmacokinetics, pharmacodynamics, and efficacy of PI3Kδ inhibitors in APDS patients. Clinical trial NCT02435173 sponsored by Novartis uses an oral PI3Kδ inhibitor leniolisib (CDZ173) (38), while clinical trial NCT02593539 sponsored by GSK uses an inhaled PI3Kδ inhibitor nemiralisib (GSK2269557) (39) that had been originally developed for treatment of COPD (34). Recently, the clinical trial NCT02435173 has reported efficacy data from six APDS patients (37). The patients were part of a 12-week within subject dose-escalation study of oral leniolisib, administered twice daily. Leniolisib was well tolerated and the study reported normalization of circulating transitional and naïve B cells, reduction in senescent T cells, decrease in the elevated serum IgM levels, and inflammatory markers. After 12 weeks of treatment, lymph node and spleen sizes reduced by 39% and 40%, respectively (37). Normalization of immunophenotypes was most notable in the final 4-week dosing period. The study has now proceeded to a long-term treatment arm with patients receiving treatment for over 9 months (70-mg leniolisib, twice daily) and no significant adverse events have been detected (37). These exciting initial findings validate the focused approach to target the activated PI3Kδ in APDS patients. It will be of interest to see if the oral or inhaled inhibitors under development provide specific advantages for the APDS patients. Inhaled PI3Kδ inhibitors will have a different safety profile and may be appropriate for patients who are primarily affected by airway infections, potentially limiting progression of bronchiectasis.

## Future Directions

Whole-exome and whole-genome sequencing of PID patients will likely identify novel variants in the *PIK3CD* and *PIK3R1* genes and it remains essential to distinguish pathogenic mutations from neutral variants. Given that APDS is a rare monogenic disorder with high penetrance, variants that cause it are unlikely to be found in healthy subjects outside of patients’ families. Therefore, excluding variants detected in healthy cohorts, e.g., reported in the gnomAD database (21), will help initial screening of potential APDS-causing mutations. However, rare variants can still be neutral, so it will remain important to demonstrate that a novel candidate mutation leads to increased PI3K activity, e.g., by showing increased levels of PIP_3_ or phosphorylated AKT. The growing list of known APDS mutations will facilitate genetic diagnosis in future patients. Early diagnosis of APDS will be essential, as it will allow early therapy, e.g., HSCT or treatment with PI3Kδ inhibitors, which should prevent many APDS complications.

As our understanding of APDS improves, new questions emerge. With more APDS patients carrying various mutations being identified, it will be interesting to understand if specific mutations are associated with disease severity and clinical or immunological subphenotypes. The varying degree of disease severity in APDS patients raises the question as to whether rare activating mutations in genes encoding PI3Kδ or other proteins that regulate PI3K activity may be responsible for causing similar disorders, perhaps resembling only some of the APDS manifestations. In support of this hypothesis, loss-of-function mutations have been described in PTEN resulting in an APDS-like phenotype (10). Larger exome- or genome-sequencing studies in patients with diseases that resemble aspects of the APDS phenotype will be interesting to explore in this regard. These studies may reveal monogenic etiology in some of the patients with disorders, such as bronchiectasis. Furthermore, it is plausible that combinations of common polymorphisms in genes regulating PI3Kδ signaling may lead to its increased activity. Such subjects may be predisposed to APDS-like manifestations, e.g., bacterial respiratory infections, herpes virus infections, or bronchiectasis. Future genetic, biochemical, and immunological studies should address these questions.

In conclusion, the story of APDS illustrates how modern biomedical approaches led to the discovery of disease etiology in a group of uncharacterized patients and then provided a novel knowledge-based therapeutic strategy. Promising data emerging from the ongoing clinical trials of PI3Kδ inhibitors (37) rises the hope that the success of this approach may translate into therapies for APDS and, possibly, for APDS-like diseases in future.

## Author Contributions

DM wrote the first draft of the manuscript and prepared Figure 1. SN edited the manuscript and prepared Table 1.

## Conflict of Interest Statement

DM is an employee of GSK. SN is a recipient of a grant from the UK Medical Research Council and GSK to study APDS.
